# The impact of sustained hyperglycemia, metabolic memory and hba1c variability in the development of chronic complications in patients with type 1 diabetes

**DOI:** 10.1186/1758-5996-7-S1-A29

**Published:** 2015-11-11

**Authors:** Larissa Carolina Garcia Franco da Rosa, Marcus Miranda, Joana Dantas, Debora Baptista Araújo, Marcus Vinicius Pinto, Leticia Maria Alcantara Margallo, João Batista Jornada Ben, Melanie Rodacki, Lenita Zajdenverg

**Affiliations:** 1 Universidade Federal do Rio de Janeiro, Rio de Janeiro, Brazil

## Background

Sustained hyperglycaemia has been linked to the development of chronic complications of type 1 diabetes (T1D) in most populations, especially if it occurs in the first few yrs. of the disease. However, this has been poorly studied in the Brazilian multi-ethnic population.

## Objectives

To assess if there is an association between the development of chronic complications (retinopathy-DR, nephropathy-DN, peripheral neuropathy– PN and cardiac autonomic neuropathy-CAN) in patients with T1D and 1) the mean glycated Haemoglobin during their follow-up since diabetes onset (mHbA1c); 2) the standard deviation (SD) of HbA1c over this period, 3) the HbA1c in the first 3 yrs. of disease (1st 3 yr.) and 4) the current HbA1c.

## Materials and methods

This retrospective study included patients with T1D ≥5 yrs. that were followed in a specialized center. Epidemiological, clinical and laboratory data were obtained by reviewing the medical charts. mHbA1c, HbA1c in the 1st 3 yr of T1D and SD of HbA1c were calculated. DR was evaluated by ophthalmoscopy. Increased urinary albumin excretion (IUAE) was defined according to the ADA criteria. CAN was diagnosed through a Questionnaire and Variability of heart rate tests. PN was defined by Neuropathy Symptoms Score and Neuropathy Disability Scores.

## Results

199 patients were assessed (54.7% women) with mean age and T1D duration of 27.9±9.7, 17.1±7.3 yrs., respectively. Their mean mHbA1c was 8.38±1.58%. DR, IUAE, PN and CAN were seen in 10.8% (12/111), 17.7% (33/188), PN in 35% (23/64) and CAN in 31.3% (33/107). Patients with IUAE had higher mHbA1c levels than others (8.95±1.53 vs. 7.99±1.18; p=0.010). MhbA1c also differed between those with PN and others (9.12±1.82 vs. 7.65±0.93; p=0.001). Although there was no association between mHbA1c and DR, the mean HbA1c in the last 10 yrs. was higher in those with DR than others (7.98±1.25 vs. 9.68±1.85; p=0.001). The SD of HbA1c was also higher in those with DR and CAN than those without these complications (p=0.004 and p=0.003, respectively). The HbA1c 1st 3yr. was higher in patients with DR (12.41±5.06 vs. 7.33±0.86; p=0.001) and in those with DN (9.35±0.45 vs. 7.71±0.81; p=0.036) than others. Current HbA1c (8.35±1.56) was not associated with either of these complications.

## Conclusions

Sustained hyperglycemia, especially in the first years of the disease, and HbC1c variability over time have been linked to the development of chronic complications in our population.

